# Cerebral *Aspergillus penicillioides* infection mimicking schwannoma in an immunocompetent host: case report with a review of the literature

**DOI:** 10.1590/1678-9199-JVATITD-2025-0091

**Published:** 2026-06-01

**Authors:** William Kazunori Sekiguchi, Juliana Possatto Fernandes Takahashi, Jessica de Brito Ferreira Nascimento, Camila da Silva Santos Ferreira, Matheus Polly, Wilker Dias Martins, Bruno Evangelista de Toledo, Thales Bhering Nepomuceno, Vinicius Trindade Gomes da Silva, Ana Catharina de Seixas Santos Nastri, Amaro Nunes Duarte, Marcello Mihailenko Chaves Magri

**Affiliations:** 1 Division of Infectious and Parasitic Diseases, Hospital das Clínicas (HC), School of Medicine (FMUSP), University of São Paulo (USP), São Paulo, SP, Brazil.; 2 Quantitative Pathology Unit, Adolfo Lutz Institute (IAL) , State Health Secretariat, São Paulo, SP, Brazil.; 3 Department of Pathology, School of Medicine (FMUSP), University of São Paulo (USP), São Paulo, SP, Brazil.; 4 Division of Neurosurgery, Hospital das Clínicas (HC), School of Medicine (FMUSP), University of São Paulo (USP), São Paulo, SP, Brazil.; 5 Central Laboratory Division, Hospital das Clínicas (HC), School of Medicine (FMUSP), University of São Paulo (USP), São Paulo, SP, Brazil.

**Keywords:** Aspergillus penicillioides, Cerebral aspergillosis, Hyalohyphomycosis, Immunocompetent host, Neoplasm mimic

## Abstract

**Background:**

Central nervous system aspergillosis is a rare and severe condition with high mortality and frequent diagnostic delay. Infection due to *Aspergillus penicillioides* in this setting is rarely reported.

**Case presentation:**

We describe a rare case of cerebral infection caused by *A. penicillioides* in an immunocompetent patient, initially mistaken for a schwannoma. Histopathological examination revealed hyaline septate hyphae, and molecular analysis of formalin-fixed and paraffin-embedded (FFPE) tissue supported the identification by sequencing of a fragment of the 28S rRNA gene. Following an initial surgical resection, clinical deterioration ensued, necessitating a repeat procedure and targeted antifungal therapy, which ultimately led to disease stabilization and outpatient discharge.

**Conclusion:**

This case highlights the diagnostic difficulties and therapeutic challenges posed by rare *Aspergillus* species in immunocompetent hosts.

## Background

*Aspergillus* species are ubiquitous environmental molds capable of causing diseases that range from allergic syndromes to life-threatening invasive infections [1]. Invasive aspergillosis classically affects immunocompromised individuals, including those with hematologic malignancies, solid organ or stem cell transplantation, or prolonged corticosteroid exposure [1, 2]. Although *Aspergillus fumigatus* (*A. fumigatus*) remains the most frequent pathogen, other species, including *A. flavus*, *A. terreus*, and *A. niger*, are increasingly recognized [3]. Among its manifestations, central nervous system (CNS) involvement remains one of the most devastating, with mortality rates exceeding 80% despite antifungal advances [3]. The infection typically results from hematogenous dissemination from a pulmonary focus or direct extension from adjacent structures, such as the paranasal sinuses or orbits [1-5]. Its clinical presentation is often subtle, and radiological findings may mimic neoplastic or granulomatous lesions, leading to significant diagnostic delay [4-6].

*A. penicillioides* is a xerophilic, extremotolerant fungus commonly isolated from dry environments and seldom associated with human infection [7, 8]. To contextualize this case, a literature search was performed across PubMed, Scopus, Web of Science, and SciELO using the search terms “*Aspergillus*” and “*penicillioides*”, which revealed only two previous reports of CNS involvement (Figure 1). Therefore, we describe a case of cerebral aspergillosis caused by *A. penicillioides* in an immunocompetent patient, initially misdiagnosed as a schwannoma, highlighting the diagnostic challenges and expanding the recognized pathogenic potential of rare *Aspergillus* species in the CNS.

**Figure 1.**
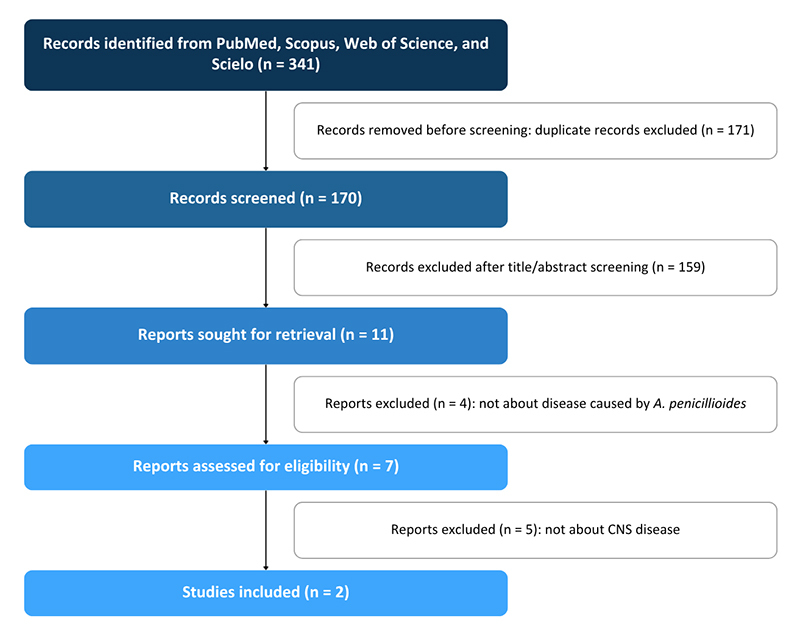
Study selection for the systematic review of *Aspergillus penicillioides* infections involving the central nervous system (CNS).

## Case presentation

A 68-year-old male farmer with frequent soil and compost exposure on his rural property presented with progressive left malar pain and hypoesthesia in the left trigeminal nerve distribution, with symptoms that begun approximately two years earlier. His medical history included hypertension, Roux-en-Y gastric bypass, and former tobacco use. After multiple visits to emergency departments, he was referred to our service for further evaluation. Brain magnetic resonance imaging (MRI) (Figure 2) performed at that time revealed a left extra-axial expansile lesion, suggestive of a schwannoma, adjacent to the ipsilateral carotid artery. The patient underwent subtotal resection a few weeks later. Initial pathology suggested a traumatic neuroma.

**Figure 2.**
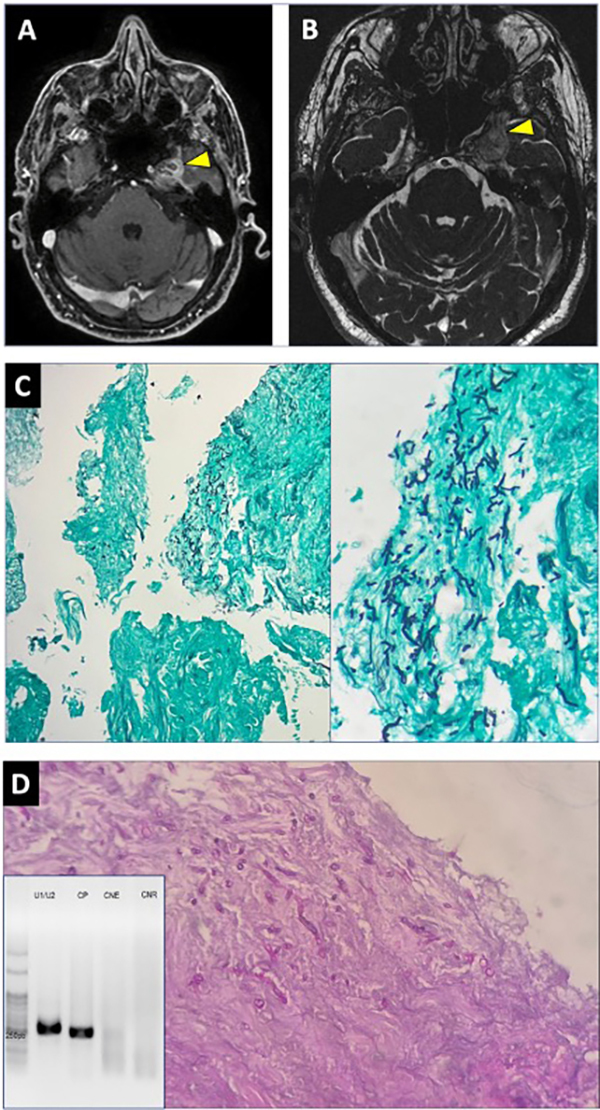
(A) Axial FIESTA-sequence brain MRI image showing an extra-axial lesion centered inside the left Meckel’s cave and insinuating through the foramen rotundum (arrowhead). (B) Axial post-contrast T1-weighted image showing a heterogeneous contrast-enhanced lesion in intimate contact with the ascending segment of the left cavernous internal carotid artery. (C) Fragment of tissue stained with Grocott’s methenamine silver showing thin, hyaline, septate hyphae with dichotomous acute-angle branching, suggestive of hyalohyphomycosis. (D) Fragment of tissue stained with Periodic Acid-Schiff (PAS) showing thin, septate hyphae with acute-angle branching, consistent with hyalohyphomycosis. Electrophoresis was performed using a Gel Doc imaging system (Bio-Rad) to visualize the specific amplicon sizes, revealing a fragment generated with the U1/U2 primers targeting the 28S rRNA gene 260 bp.

Postoperatively, he continued to experience severe pain, and about eight months later, he developed diplopia, convergent deviation of the left eye, and low-grade fevers. Repeat brain MRI (Figures 3A-3C ) demonstrated recurrence and enlargement of the residual lesion, now involving the cavernous sinus and encasing the left internal carotid artery. Serial pathology reviews raised suspicion for filamentous fungal infection (Figures 2C-2D ).

**Figure 3.**
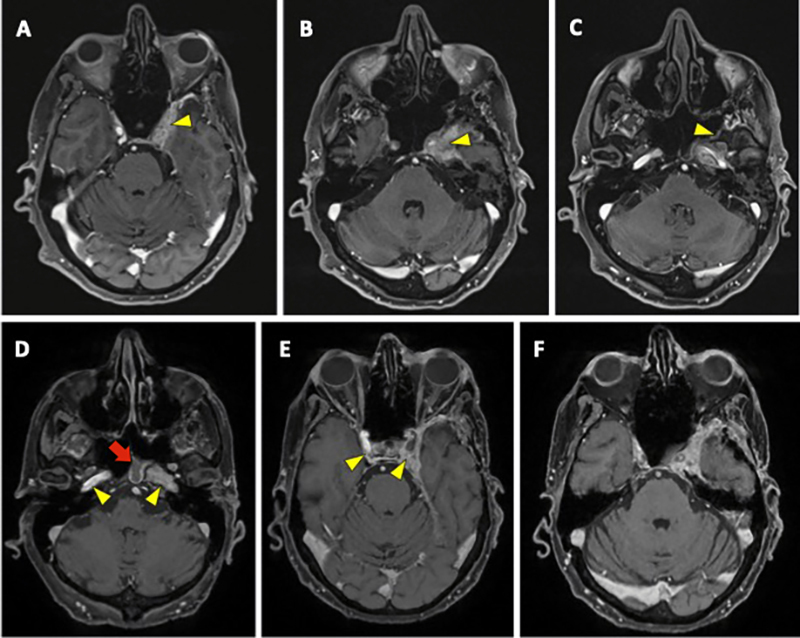
Magnetic resonance imaging findings of cerebral aspergillosis caused by *Aspergillus penicillioides*. **(A)** Axial post-contrast T1-weighted MRI showing a lesion infiltrating the left cavernous sinus after the first intracranial surgical procedure. **(B)** Axial post-contrast T1-weighted MRI demonstrating progression of the previously described lesion within the left Meckel’s cave. **(C)** Axial post-contrast T1-weighted MRI showing circumferential involvement of the petrous segment of the left internal carotid artery, accompanied by mild luminal stenosis at the same level (arrowhead). **(D)** Axial post-contrast T1-weighted MRI showing the previously described contrast-enhancing lesion at the left petrous apex, now demonstrating progression with worsening stenosis of the petrous segment of the left internal carotid artery and absence of intraluminal contrast filling (arrowheads; compare with contralateral vessel). Discontinuity of the lateral sphenoid sinus wall at the same level, suggestive of local infiltration, and associated mucosal thickening are also observed (red arrow). **(E)** Axial post-contrast T1-weighted MRI demonstrating a lesion occupying the left cavernous sinus, associated with absence of contrast filling in the cavernous segment of the left internal carotid artery. **(F)** Axial post-contrast T1-weighted MRI showing the lesion inside Meckel’s Cave.

The patient was rehospitalized and underwent a repeat subtotal resection. He received liposomal amphotericin B (L-AmB) at 5 mg/kg, as mucormycosis could not be excluded. Intraoperative cultures were negative for bacteria and fungi; however, histopathology revealed septate hyphae suggestive of *Aspergillus* spp. Cerebrospinal fluid analysis revealed normal biochemistry and was negative for bacteria, mycobacteria, and fungi. Further evaluation demonstrated a serum galactomannan (GM) index of 0.12, negative *Aspergillus* and HIV serologies, and a chest computed tomography (CT) without abnormalities.

Formalin-fixed and paraffin-embedded (FFPE) tissue obtained during the surgical procedure was subjected to molecular analysis. DNA was extracted using the GeneJET™ FFPE DNA Purification Kit (Thermo Fisher Scientific, Waltham, MA, USA), according to the manufacturer’s instructions. Conventional polymerase chain reaction (cPCR) targeting the 28S rRNA gene was performed using the primers U1 (5′-GTGAAATTGTTGAAAGGGAA-3′) and U2 (5′-GACTCCTTGGTCCGTGTT-3′), as previously described [9]. The reaction mixture consisted of 1.0 µL of each primer (10 µM), 12.5 µL of GoTaq^®^ Green Master Mix (Promega Corporation, Madison, WI, USA), containing 400 µM of each dNTP and 3 mM MgCl₂, 0.5 µL of nuclease-free water, and 10 µL of extracted DNA, for a final volume of 25 µL.

Amplification was performed in a VeritiPro™ Thermal Cycler (Applied Biosystems™, Thermo Fisher Scientific, Waltham, MA, USA) with the following cycling conditions: initial denaturation at 94 °C for 10 minutes; 45 cycles of denaturation at 94 °C for 1 minute, annealing at 58 °C for 1 minute, and extension at 72 °C for 1 minute; followed by a final extension at 72 °C for 10 minutes. A positive control, an extraction negative control, and a reaction negative control were included. PCR products were resolved by electrophoresis on a 1.5% agarose gel and visualized using a Gel Doc™ XR+ System (Bio-Rad Laboratories, Hercules, CA, USA). Amplicons of approximately 260 bp were purified and subjected to bidirectional Sanger sequencing on an ABI 3500 Genetic Analyzer (Applied Biosystems, Foster City, CA, USA) using the BigDye™ Terminator v3.1 Cycle Sequencing kit. Chromatograms were edited using Sequencher® version 4.1.4 (Gene Codes Corporation, Ann Arbor, MI, USA) and inspected with FinchTV version 1.4.0 (Geospiza Inc., Seattle, WA, USA). BLAST analysis (National Center for Biotechnology Information, NCBI) and comparison with MycoBank reference sequences revealed the highest similarity to *A. penicillioides*. The sequence showed 100% query coverage with 92-93% identity values against deposited reference sequences. Following analysis, the sequence was deposited in GenBank under submission number SUB15620711.

After 18 days of L-AmB, intravenous voriconazole (VCZ) was initiated when histopathology confirmed hyphae suggestive of *Aspergillus* spp. VCZ was administered as a loading dose of 6 mg/kg every 12 hours for two doses, followed by 4 mg/kg every 12 hours. Follow-up brain MRI (Figures 3D-3F ), two months after the second surgery, showed stable fungal disease with severe focal stenosis of the left internal carotid artery.

Given the high surgical risk of carotid resection, including potential ischemic complications and mortality, he was discharged on oral antifungal therapy. Considering his history of Roux-en-Y gastric bypass and the possibility of impaired VCZ absorption, oral isavuconazole (ISA) was chosen for outpatient management.

## Discussion

This report documents the third known case of *A. penicillioides* infection involving the central nervous system. After a literature review across the major databases (PubMed, Scopus, Web of Science, and SciELO) using the search terms *Aspergillus* AND *penicillioides*, a total of 341 articles were identified. Of these, 171 duplicates were removed, and 159 articles were excluded after abstract screening. Eleven articles underwent full-text review, among which we identified seven reporting human disease. Of these, only two [10, 11] described involvement of the central nervous system (Figure 1). The first case involved a 3-month-old boy with suspected cystic fibrosis who presented to the hospital with respiratory failure following a chronic course of cough. During hospitalization, his condition progressively deteriorated, culminating in multiorgan failure and death. Autopsy revealed disseminated *A. penicillioides* infection involving the lungs, heart, kidneys, bladder, gastrointestinal tract, and brain. The second case concerned a 38-year-old woman who, following neurosurgical resection of an acoustic neuroma (vestibular schwannoma), developed fever and a rapidly worsening clinical status 15 days after the procedure. The patient died, and an autopsy identified the rupture of a fungal aneurysm of the posterior inferior cerebellar artery caused by *A. penicillioides*. Table 1 presents a comparison between the two reported cases and our patient.

**Table 1.** Comparison of our case and the literature case reports of *Aspergillus penicillioides* central nervous system infection.

Author	Patient	Clinical presentation	Diagnosis	Site of involvement	Antifungal treatment	Outcome
Gupta et al. **[** 10 **]**	3-month-old infant boy, with suspected cystic fibrosis	Respiratory distress, lethargy	Post-mortem	Lungs, kidneys, bladder, gastrointestinal tract, heart, brain	None	Death
Tambuzzi et al. **[** 11 **]**	38-year-old woman, with acoustic neurinoma	Worsening fatigue, fever	Post-mortem	Brain	None	Death
Sekiguchi et al. [present study]	68-year-old man, with hypertension, Roux-en-Y gastric bypass	Progressive malar pain, hypoesthesia in trigeminal nerve distribution, diplopia, fever	Histopathological examination, and molecular testing	Brain	18 days of L-AmB, 18 days of VCZ, 2 months of ISA	Outpatient discharge

L-AmB: liposomal amphotericin B; VCZ: voriconazole; ISA: isavuconazole

Traditionally regarded as a xerophilic environmental mold with minimal pathogenic potential, *A. penicillioides* has only rarely been implicated in superficial or ocular infections [7, 8, 12]. Its recovery from brain tissue challenges the classical epidemiologic boundaries of invasive aspergillosis and expands the spectrum of *Aspergillus* species capable of neurotropism. The present case underscores that, even in immunocompetent individuals, chronic localized fungal invasion may occur and clinically simulate neoplastic lesions. 

Our case also emphasizes the involvement of a distinctive *Aspergillus* species. The majority of CNS aspergillosis cases are caused by *A. fumigatus*, with occasional reports involving *A. flavus*, *A. terreus*, and *A. niger* [3]. Recent taxonomic revisions have considerably expanded the understanding of the *Aspergillus* section *Restricti*, a group of xerophilic fungi adapted to environments with low water activity, such as indoor dust, stored materials, and high-salinity niches [13]. *A. penicillioides*, the most frequent species in this section, has been isolated from domestic environments, museum collections, and even deep-sea sediments, reflecting its remarkable ecological versatility. Traditionally considered a contaminant or a cause of cultural asset deterioration, it has also been implicated in rare human infections, including keratomycosis [12]. Molecular phylogenetic analyses using multilocus sequencing (ITS, *benA*, *CaM*, *RPB2*) have confirmed that *A. penicillioides* is part of a distinct clade within the *A. penicillioides* complex, closely related to *A. clavatophorus* and *A. magnivesiculatus*, yet genetically and phenotypically separable [13]. These findings underscore that members of section *Restricti*, despite their preference for dry microenvironments, can act as opportunistic pathogens under favorable host or tissue conditions, such as the cerebral infection documented here [10, 11].

CNS aspergillosis is associated with high mortality and heterogeneous clinical manifestations, most commonly headache and focal neurological deficits, depending on the lesion location. The infection typically occurs in immunocompromised hosts and may arise via hematogenous dissemination or direct extension from paranasal sinus disease [3, 5]. We describe a 68-year-old hypertensive man with a history of bariatric surgery who developed CNS aspergillosis with severe vascular involvement. The absence of sinus disease suggests hematogenous spread as the most likely mechanism. One plausible explanation for such an aggressive presentation in an immunocompetent host is exposure to a high fungal inoculum during composting activities on his rural property, highlighting the potential role of environmental factors.

The insidious clinical course and radiological features initially resembling a schwannoma resulted in a delayed diagnosis. Siddiqui et al. [14] reported a series of CNS aspergilloma cases in immunocompetent patients in which sinus disease was present in all cases. Diagnosis relied primarily on histopathology, which revealed hyphae compatible with *Aspergillus* spp., whereas culture yielded positive results in only 25% of patients [14]. Similarly, histopathological examination in the present case was essential to exclude neoplasia and raise suspicion for a filamentous fungal infection. Subsequent genetic sequencing confirmed the diagnosis, underscoring the value of molecular tools in identifying rare presentations.

Therapeutic decisions in CNS aspergillosis should integrate both surgical and pharmacologic strategies. Current guidelines recommend voriconazole as the first-line therapy because of its fungicidal activity and favorable CNS penetration [1, 2]. L-AmB remains an acceptable initial option, particularly when the diagnosis is uncertain or when resistant molds are possible.

Despite the delay in diagnosis, our patient underwent subtotal resection of the lesion and received effective antifungal therapy. Empirical treatment was initiated with L-AmB owing to the initial differential diagnosis that included mucormycosis. After confirmation of aspergillosis, therapy was switched to intravenous VCZ until discharge. The choice of ISA as the oral antifungal agent was guided by the patient's history of Roux-en-Y gastric bypass, which could impair VCZ absorption. Moreover, ISA provides excellent bioavailability and absorption after bariatric surgery [15] and is consistent with current guideline recommendations for aspergillosis management [1, 2].

This study has some limitations. Molecular identification was based on sequencing of a single ribosomal target (28S rRNA), which provides limited taxonomic resolution within *Aspergillus* section *Restricti*. Although the analysis supported the identification of *A. penicillioides*, multilocus sequencing would be required for unequivocal species-level confirmation. Therefore, the molecular findings in this case should be interpreted as supportive of the histopathological diagnosis of invasive aspergillosis rather than definitive taxonomic characterization. The choice of U1/U2 primers targeting a short fragment of the 28S rRNA gene was deliberate and based on the known limitations of DNA integrity in formalin-fixed paraffin-embedded (FFPE) tissues.

## Conclusions

In conclusion, our case highlights the diagnostic challenges of CNS aspergillosis, particularly when initial imaging suggests a neoplastic lesion. Genetic sequencing was essential for establishing the diagnosis, underscoring the importance of molecular tools in identifying rare fungal pathogens.

## Availability of data and materials

 Not applicable.
